# Inhibition of histone methyltransferase G9a promotes the osteogenic potential of bone-derived stem cells in diabetic-osteoporosis by regulating the LINC00657/miR-204-5p/IGFBP5 pathway

**DOI:** 10.3389/fendo.2025.1625944

**Published:** 2025-08-12

**Authors:** Mei-qi Qiao, Bin Wang, Jian-pin Fan, Feng Gao, Shao-jun Wang, Sheng-yang Guo, Sheng-li Xia

**Affiliations:** ^1^ Graduate School, Shanghai University of Traditional Chinese Medicine, Shanghai, China; ^2^ Department of Orthopedics, Shanghai University of Medicine & Health Sciences Affiliated Zhoupu Hospital, Shanghai, China

**Keywords:** G9a inhibitor, diabetic osteoporosis, LINC00657, miR-204-5p, IGFBP5

## Abstract

**Background:**

Bone mesenchymal stem cells (BMSCs) from patients with diabetes often exhibit reduced osteogenic potential. This study aimed to investigate the mechanism of action of G9a, known as euchromatic histone lysine methyltransferase 2 (EHMT2), identify its key responsive long non-coding RNA in diabetic osteoporosis (DOP), and evaluate the effectiveness of the G9a inhibitor (UNC0638).

**Methods:**

The expression level of G9a in bone-derived MSCs (BMSCs) from osteoporosis patients with or without T2DM (T2DM-BMSCs, CON-BMSCs) was detected, and osteogenic differentiation was evaluated by osteogenic genes, ALP activity and calcification level. The key lncRNA, LINC00657, was screened based on previous transcriptome sequencing, qPCR and gene overexpression assay. The downstream miRNA and the target gene of LINC00657 were identified through transcriptome sequencing, bioinformatics analysis, dual luciferase reporter assay and gene overexpression assay. Rat DOP was constructed, and micro-CT, histochemical staining, immunofluorescence and qPCR were used to investigate the mechanism of UNC0638.

**Results:**

G9a expression was increased and LINC00657 expression was decreased in T2DM-BMSCs, compared with CON-BMSCs. UNC0638 treatment improved the osteogenic potential of T2DM-BMSCs and reversed the downregulation of LINC00657. LINC00657 overexpression reverses the inhibitory effect of EHMT2 on osteogenic differentiation. miR-204-5p and IGFBP5 were screened as downstream molecules of LINC00657. LINC00657 was able to sponge miR-204-5p and upregulated IGFBP5 expression, thereby promoting osteogenesis in T2DM-BMSCs. UNC0638 treatment alleviated osteoporosis in DOP rats, whereas LINC00657 knockdown inhibited its effect *in vivo*.

**Conclusions:**

G9a inhibits the osteogenic potential of T2DM-BMSCs by regulating the LINC00657/miR-204-5p/IGFBP5 pathway and UNC0638 may be a potential agent for DOP treatment.

## 1 Introduction

Diabetes mellitus (DM) is a chronic metabolic disorder characterized by hyperglycemia caused by insulin deficiency or resistance. The prevalence of DM is estimated to increase to 12.2% by 2045, affecting approximately 783.2 million individuals worldwide (1). In China, approximately 30% of the elderly population has DM, with over 95% of cases being type 2 DM (T2DM) (2). Osteoporosis is a systemic skeletal disease characterized by reduced bone mass and deteriorated bone microarchitecture, leading to increased fracture risk (3). It is also a common complication of DM, with approximately 50–66% of cases displaying decreased bone mineral density (BMD) and approximately 33% diagnosed with diabetic osteoporosis (DOP) (4, 5). The pathophysiology of DOP is characterized by insulin and IGF-1 deficiency, hyperglycemia, advanced glycation end products (AGEs), pro-inflammatory cytokines, and oxidative stress (6, 7). Control of hyperglycemia, and prevention and treatment of bone loss remain major strategies, including lifestyle modifications, insulin or oral hypoglycemic agents, and anti-osteoporosis drugs (8). Nevertheless, the effects of certain hypoglycemic agents on bone metabolism are becoming increasingly recognized and their inappropriate use may increase the risk of fractures (9, 10). Epigenetic modifications, including histone modifications, DNA methylation, and non-coding RNAs, play pivotal roles in bone metabolic homeostasis, offering novel insights into potential therapeutic targets (11–13).

G9a, also known as euchromatic histone lysine methyltransferase 2 (EHMT2), is a member of the suppressor of the variegation 3-9 (su(var)3-9) family. G9a is capable of catalyzing the mono- and di-methylation of histone 3 lysine 9 (H3K9) and regulating the methylation of the cofactor S-adenosylmethionine (SAM) (14). In addition, G9a can act as a transcriptional coactivator independent of its methyltransferase activity. G9a regulates gene expression in different ways, thereby playing an important role in various biological and pathological processes (15–17). The role of G9a in DM relevant diseases and metabolism remains controversial. G9a is decreased in the livers of high-fat diet (HFD)-fed mice and upregulation of G9a prevents palmitic acid-induced insulin resistance (18). Another report indicated that the knockout of muscular G9a rendered mice resistant to HFD-induced obesity and hepatic steatosis by regulating the muscle-liver-fat metabolic axis (19). Furthermore, several G9a plays an important role in bone metabolism and differentiation. Deletion of G9a causes incomplete ossification and shorter jaws (20) and administration of the G9a inhibitor A366 decreases the osteogenic potential of bone marrow-derived mesenchymal stem cells (BMMSC) (21). The G9a inhibitor BIX01294 suppresses the osteoclast differentiation of Raw264.7 cells (22). In addition, micromolar levels of BIX01294 promote the cardiac differentiation of BMMSC (23). These studies indicate a negative role of G9a inhibitors in osteogenesis. Our previous study found that bone-derived mesenchymal stem cells (BMSC) from patients with osteoporosis and T2DM (T2DM-BMSCs) showed lower osteogenic potential than those isolated from patients with osteoporosis without T2DM (24). Other studies have also demonstrated the weaker osteogenic potential of T2DM-BMSCs (25, 26). Relevant mechanisms include cellular senescence, accelerated apoptosis and disrupted differentiation balance of BMMSC, induced by hyperglycemia (27). Additionally, activation of TLR4/NF-κB pathway and abnormal epigenetic regulation also inhibits osteogenic potential of BMMSC (28, 29). Currently, the role of G9a in T2DM-BMSCs is still unknown.

Long non-coding RNA (lncRNAs), which are more than 200 nucleotides in length, have only been considered byproducts of transcription and are assumed to lack biological effects. Nevertheless, lncRNAs play multifaceted roles in the regulation of gene expression at various levels, thereby participating in the development of osteoporosis and DM (30). Notably, lncRNAs are involved in DOP (31). LncRNA *AK137033* inhibits the osteogenic potential of adipose-derived stem cells in DOP mice by regulating DNA methylation (32). Notably, some lncRNAs, such as *MALAT1* (33) and Lnc-Rewind (34), can regulate G9a function, and the G9a inhibitor, RK-701, regulates the expression of lncRNA BGLT3 (35), indicating crosstalk between lncRNAs and G9a.

In the present study, we found that G9a expression was increased in T2DM-BMSCs, and nanomoles of the G9a inhibitor, UNC0638, improved the osteogenic potential of T2DM-BMSCs. Based on previous transcriptome sequencing, LINC00657 was screened, and its expression was found to be regulated by G9a. Next, LINC00657 was shown to promote osteogenic potential by regulating the miR-204-5p/insulin-like growth factor-binding protein 5 (IGFBP5) pathway based on bioinformatics analysis and dual luciferase reporter assay. Therefore, we hypothesized that G9a-mediated epigenetic regulation impairs the osteogenic potential of T2DM-BMSCs through modulating lncRNA LINC00657 and the miR-204-5p/IGFBP5 axis. Finally, the treatment effects of UNC0638 and its mechanism of action were confirmed in a rat model of DOP.

## 2 Materials and methods

### Cells and cell culture

The T2DM-BMSCs obtained in our previous study (24) were cultured in MEM supplemented with 10% fetal bovine serum (Gibco, CA, USA) and 1% streptomycin/penicillin (Gibco). The 293T and L929 cells (mouse fibroblasts), purchased from the Cell Bank of the Chinese Academy of Sciences (Shanghai, China), were cultured in DMEM (Hyclone) supplemented with the same supplements. All cells were maintained at 37°C in a 5% humidified CO_2_ atmosphere.

### Quantitative PCR

Total RNA of cells or bone tissue was extracted using a FastPure^®^ Cell/Tissue Total RNA Isolation Kit (Vazyme, Nanjing, China) and 1 μg RNA was used for cDNA synthesis by using a HiScript^®^ III RT SuperMix for qPCR (Vazyme). The ChamQ SYBR qPCR Master Mix (Vazyme) was used for qPCR. GAPDH was used as the internal control for mRNA and lncRNA expression. The MiPure Cell/Tissue miRNA Kit (Vazyme) was used to extract total miRNA. The miRNA 1st Strand cDNA Synthesis Kit (Vazyme) and MiRNA Universal SYBR qPCR Master Mix (Vazyme) were used for cDNA synthesis and qPCR, respectively. U6 was used as an internal control for miRNAs. All procedures were performed according to the manufacturer’s instructions. Relative expression level of gene was calculated using the 2^−ΔΔCt^ method. The primer sequences used for qPCR and miRNA reverse transcription are listed in **Supplementary Table S1**.

### Western blotting

Cells were lysed using RIPA lysis buffer (Beyotime, Shanghai, China), and protein concentration was quantified using a BCA kit (Beyotime). SDS-PAGE (10%) was used to isolate the denatured protein (20 μg), which was then transferred into the 0.22μm PVDF membrane. The membrane was then incubated with protein-free rapid blocking buffer (Servicebio, Wuhan, China) for 5 min at room temperature. After washing with TBST for 2 min, the membranes were incubated with corresponding antibody at 4°C overnight, followed by 1.5 h-incubation with the secondary antibody at room temperature. The bands were visualized using an ECL detection system (Beyotime). The antibodies: GAPDH, EHMT2/G9a, GLP, H3K9me1, H3K9me2, H3, RUNX2, BGLAP and COL1A1 were purchased from ABclonal Biotechnology Co., Ltd. (Wuhan, China), and diluted at a ratio of 1:1000 prior to utilisation.

### Cytotoxicity

Cells were seeded in 96-well plates at a density of 4000 cells/well. Different concentrations (0, 0.1, 0.5, 1, 5, 10, 25, 50, and 100 μM) of UNC0638 (Medchemexpress, Shanghai, China) were incubated with cells for 3 or 7 days respectively. Then, 5 mg/ml MTT (Sigma-Aldrich, Shanghai, China) was incubated with the cells for 3 h, crystal violet was dissolved in DMSO (Sigma-Aldrich), and the optical density was detected at 492 nm using a microplate reader.

### ALP staining and activity detection

After 7 days of osteogenic induction, as described in a previous study (24), the cells were fixed and stained with BCIP/NBT stain (Beyotime) for 1 h following the manufacturer’s protocols. An ALP detection kit (Beyotime) was used to detect ALP activity according to the manufacturer’s instructions.

#### Alizarin red S staining

After 14 days of osteogenic induction, the cells were fixed and stained with ARS reagent (Solarbio, Beijing, China) according to the manufacturer’s protocol. Subsequently, 10% cetylpyridinium chloride was used to dissolve the calcified matrices and the optical density was measured at 562 nm.

### Cell transfection

The cDNA sequences of EHMT2, LINC00657, or IGFBP5 were amplified and subcloned into a pcDNA3.1 vector. An empty vector was used as the negative control (NC). Overexpression vectors for EHMT2, LINC00657, or IGFBP5 were transfected into the cells as previously described (24). miR-204-5p and NC mimics were synthesized by GenePharma (Shanghai, China), and transfected into cells using Lipofectamine 2000 (Invitrogen, Thermo Fisher Scientific) following the manufacturer’s instructions. After 48 h, cells were collected for qPCR analysis.

### Transcriptome sequencing

Cells overexpressing the LINC00657 overexpression vector or the NC vector were prepared, and total RNA was extracted using TRIzol (Invitrogen), then purified using an RNeasy Mini Kit (Qiagen, Germany). The samples were subjected to cDNA library construction and sequencing by Sinotech Genomics Co. Ltd. (Shanghai, China). The R package edgeR was used to analyze differential gene expression. |Fold change (FC)| value > 2 and p-value < 0.05 were used for screening differentially expressed genes (DEGs).

### Bioinformatics analysis

MiRNAs of LINC00657 were predicted using LncBase Predicted v.2 (36) and a score ≥ 0.8 was used as the filter. TargetScan (http://www.targetscan.org/vert_72/) was used to predict the miRNAs of candidate genes, and score percentile ≥ 80% was used as the threshold. The ceRNA network of LINC00657 was constructed using the Cytoscape software (version 3.5.1). GO analysis of biological processes, cellular components, and molecular functions was performed using the enriched R package.

### Dual luciferase reporter assay

The 3′-UTR of LINC00657-WT, LINC00657-MUT, IGFBP5-WT, and IGFBP5-MUT was amplified using PCR and inserted into the pmirGLO vector (Invitrogen, CA, USA). LINC00657-WT or LINC00657-MUT vectors, IGFBP5-WT or IGFBP5-MUT vectors, and miR-204-5p or NC mimics were co-transfected into 293T cells using Lipofectamine 3000 (Invitrogen). Forty-eight hours post-transfection, dual-luciferase reporter gene assays were performed to measure luciferase activity using a Dual-Luciferase Reporter Assay System kit (Promega, USA) according to the manufacturer’s instructions.

### Construction of recombinant AAV2-U6-sh-LINC00657-EGFP

pAAV-U6-sh-LINC00657-EGFP, constructed by GenePharma (Shanghai, China), and packaging plasmids (pHelper and pAAV2, GenePharma) were co-transfected into 293T cells to produce recombinant AAV2 (AAV2-U6-sh-LINC00657-EGFP, AAV2/sh-LINC00657), as described in a previous study (37). A blank AAV (AAV2/sh-NC) was produced using the same method.

### Rat DOP model and treatments

Twenty-five specific-pathogen-free male SD rats (220 ± 20 g), purchased from Shanghai Lab Animal Research Center (Shanghai, China), were randomly divided into a normal diet group (control, n=5) and high glucose and high fat (HGHF) diet group (n=20). Rats in the HGHF group received the HGHF diet for 12 weeks, followed by an intraperitoneal injection of STZ (Sigma-Aldrich) at a dose of 30 mg/kg (38). Animals with blood glucose levels >16.7 mmol/L for 3 days were considered T2DM models. Blood glucose levels were measured using a glucose meter (Omron, Shanghai, China). The control rats were fed a normal diet and intraperitoneally injected with normal saline. In addition, all rats were maintained under appropriate conditions (20–25°C with 65–80% humidity) with free access to water and food.

Next, the T2DM rats were randomly divided into four groups. Model group (n=5): intraperitoneal injection of normal saline; UNC0638 group (n=5): intraperitoneal injection of UNC0638 (5 mg/kg) twice a week for 12 weeks, according to previous studies (39, 40) with some modifications; UNC0638 + sh-NC group (n=5): intraperitoneal injection of UNC0638 (5 mg/kg) twice a week for 12 weeks, AAV2/sh-NC (3 × 10^12^ vg/kg, 50 μl) (41); and UNC0638 + sh-LINC00657 group (n=5): intraperitoneal injection of UNC0638 (5 mg/kg) twice a week for 12 weeks, with intravenous injection of AAV2/sh-LINC00657 (3 × 10^12^ vg/kg, 50 μl). All rats with T2DM were fed an HGHF diet for 12 weeks. At the end of the assay, all rats were anesthetized using intraperitoneal injection of chloral hydrate (400 mg/kg) and euthanized using cervical dislocation. Bilateral femurs were isolated for further analysis. This animal study was approved by the Ethics Committee of Zhoupu Hospital (ZPYYLL-018-02). All procedures were performed in accordance with the guidelines of the Care and Use of Laboratory Animals (Ministry of Science and Technology of China, 2006).

### Serum ALP and OCN detection

The blood of rats was collected, and the serum was obtained after centrifugation at 1,100×g for 10 min at 4°C. ALP and OCN levels were detected using a rat alkaline phosphatase ELISA kit (Elabscience, Wuhan, China) and osteocalcin ELISA kit (Elabscience), respectively, following the manufacturer’s instructions.

### Micro-CT analysis

The femurs of rats were collected and fixed with 4% paraformaldehyde for 24 h, and the distal femur was scanned using an X-ray microtomography imaging system (SkyScan1276, Bruker, Germany). Trabecular bone volume (BV/TV; %), trabecular thickness (Tb.Th; mm), BMD (mg/cm^2^), structure model index (SMI), trabecular number (Tb.N; mm^–1^), bone surface/volume ratio (BS/BV; mm^–1^), and trabecular separation (Tb.Sp; mm) were evaluated.

### Immunohistochemistry

After fixation, decalcification, embedding, sectioning, and deparaffinage, the slices of distal femurs were blocked with 10% goat serum, and incubated with G9a (1:200, ABclonal), OPN (1:200, ABclonal), and COL1A1 (1:200, ABclonal) at 4°C overnight. The sections were then incubated with a secondary antibody (ABclonal) for 2 h at room temperature, followed by nuclear staining with hematoxylin (Servicebio). The IHC results were visualized using a microscope (Olympus, Tokyo, Japan).

### Hematoxylin and eosin staining and Masson’s staining

The prepared slices of the distal femurs were stained with H&E (Servicebio) or Masson’s (Servicebio)dye, as previously described (42), and then visualized using a microscope (Olympus).

### Statistical analysis

The experimental data were expressed as mean ± standard deviation and analyzed using SPSS 17.0 software (SPSS, Inc., Armonk, USA). The unpaired Student’s t-test was used to analyze the differences between two groups. One-way analysis of variance (ANOVA) with Tukey’s *post hoc* test was used to compare multiple groups. P<0.05 was considered statistically significant.

## 3 Results

### UNC0638 promoted the osteogenic differentiation of T2DM-BMSCs

**Figure 1:**
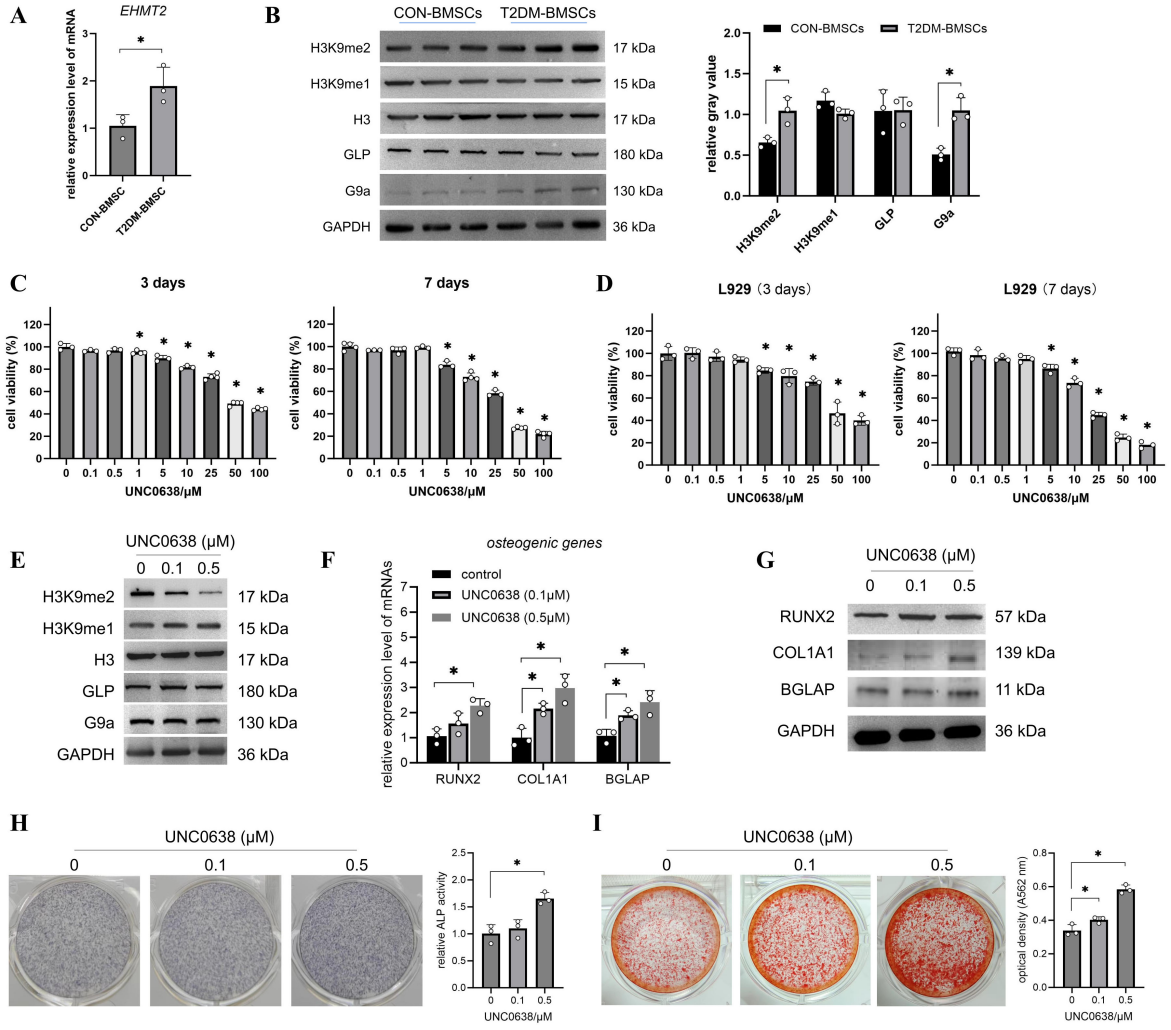
UNC0638 promoted the osteogenic differentiation of T2DM-BMSCs. **(A)** qPCR detection of *EHMT2* mRNA levels in T2DM-BMSCs and CON-BMSCs. **(B)** Western blotting of G9a, GLP, H3K9me2, and H3K9me1 protein levels in T2DM-BMSCs and CON-BMSCs (n=3). **(C,D)** T2DM-BMSCs **(C)** or L929 cells **(D)** were treated with UNC0638 for 3 or 7 days, and MTT was performed to detect cell viability. **(E)** Western blotting of G9a, GLP, H3K9me2, and H3K9me1 protein levels in T2DM-BMSCs treated with UNC0638. **(F–I)** T2DM-BMSCs underwent osteogenc induction for 7 days with the addition of UNC0638, were induced for 7 days, osteogenic genes and proteins (*RUNX2, COL1A1*, and *BGLAP*) were detected using qPCR **(F)** and western blotting **(G)**, ALP activity was detected using staining and activity quantification **(H)**; mineralization level was detected using ARS staining following 14 days of induction **(I)**. BMSCs, bone marrow-derived mesenchymal stem cells; T2DM, type 2 diabetes mellitus. *, P<0.05.

As shown in **Figure 1A**, the *EHMT2* mRNA and G9a protein expressions were significantly enhanced in T2DM-BMSCs compared with those in the controls (**Figure 1B**, p < 0.05). The protein levels of GLP and H3K9me1 exhibited minimal alterations, while H3K9me2 level demonstrated a notable increase in T2DM-BMSCs (**Figure 1B**, p < 0.05). The alterations in EHMT2 and H3K9me2 expression may be related to the weaker osteogenic differentiation potential of T2DM-BMSCs. Next, the cytotoxicity of UNC0638, a G9a inhibitor, in BMSCs was evaluated. The MTT result indicated that, 0.1–0.5 μM of UNC0638 was non-cytotoxic on T2DM-BMSCs after treatment for 3 or 7 days, and the cell viability reduced by 40–80% when the dose was ≥ 25 μM (**Figure 1C**). For the normal cells (L929), the safe dose of UNC0638 was 0.1-1 μM (**Figure 1D**), higher than that observed in the T2DM-BMSCs. Then the safe doses (0.1 and 0.5 μM) were used for further detection. Treatment with 0.1 or 0.5 µM of UNC0638 gradually reduced H3K9me2 levels, without affecting G9a, GLP, or H3K9me1 (**Figure 1E**). As demonstrated in **Figures 1F–I**, treatment with UNC0638 resulted in a significant enhancement of *RUNX2*, *COL1A1*, and *BGLAP* expression and protein levels, as well as ALP activity and calcification levels. In addition, 0.5 μM of UNC0638 showed a better promotion and significance than that at 0.1 μM.

### Overexpression of LINC00657 reversed the inhibitory role of EHMT2 on osteogenic differentiation

**Figure 2:**
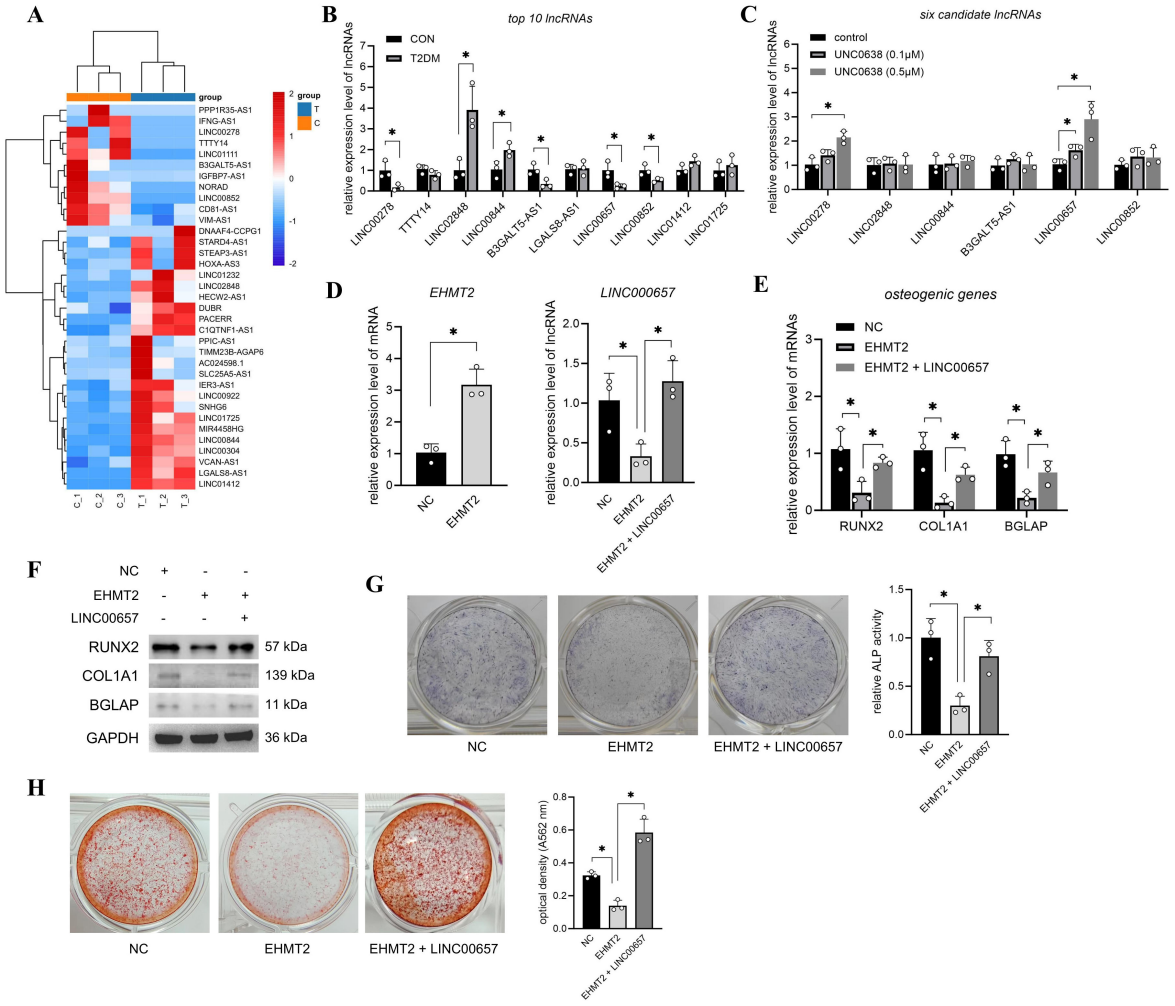
Overexpression of LINC00657 reversed the inhibitory role of EHMT2 on osteogenic differentiation. **(A)** Heatmap of 35 known significantly changed lncRNAs in T2DM-BMSCs. T: T2DM group, C: CON group. **(B)** qPCR detection of top 10 lncRNAs in T2DM group (vs CON group). **(C)** qPCR detection of six candidate lncRNAs after UNC0638 treatment (vs CON group). **(D)** T2DM-BMSCs were transfected with EHMT2 overexpression vector or co-transfected with LINC00657 overexpression vector, and qPCR was performed to detect expression levels of EHMT2 and LINC00657. **(E–H)** T2DM-BMSCs overexpressed with EHMT2 (and LINC00657) were induced for 7 days, osteogenic genes and proteins (*RUNX2, COL1A1*, and *BGLAP*) were detected using qPCR **(E)** and western blotting **(F)**. ALP activity was detected using staining and activity quantification **(G)**; mineralization level was detected using ARS staining following 14 days of induction **(H)**. BMSCs, bone marrow-derived mesenchymal stem cells; T2DM, type 2 diabetes mellitus. *, P<0.05.

Previous transcriptome sequencing detected thousands of lncRNAs in T2DM-BMSCs and 88 significantly altered lncRNAs (FC > 2, p < 0.05) were screened, including 35 known and 53 unknown lncRNAs (**Supplementary Table S2**). **Figure 2A** shows the expression profiles of 35 known lncRNAs in each cell sample. The top 10 lncRNAs were selected for qPCR validation. As shown in **Figures 2B, C**, six lncRNAs (*LINC00278, LINC02848, LINC00844, B3GALT5-AS1, LINC00657*, and *LINC00852*) were significantly altered in T2DM-BMSCs (p < 0.05), and UNC0638 treatment caused significant upregulation of *LINC00278* and *LINC00657* [also known as NORAD (43)] (p < 0.05). Therefore, UNC0638 reversed the downregulation of these two lncRNAs in T2DM-BMSCs.

Next, LINC00657, which has a higher sensitivity to UNC0638 than that of LINC00278, was selected to investigate its role in G9a-mediated osteogenic differentiation. qPCR detection confirmed that *EHMT2* mRNA levels were significantly enhanced after T2DM-BMSCs transfection with the EHMT2 overexpression vector (p < 0.05) and overexpression of EHMT2 caused a decrease in *LINC00657* (**Figure 2D**), consistent with the results regulated by UNC0638. LINC00657 expression was enhanced after T2DM-BMSCs were co-transfected with the LINC00657 overexpression vector (**Figure 2D**). In addition, overexpression of EHMT2 inhibited the mRNA and protein levels of *RUNX2, COL1A1*, and *BGLAP*, as well as ALP activity and calcification levels, whereas upregulation of LINC00657 partially restored osteogenic marker expression, ALP activity and mineralization capacity (**Figures 2E–H**). These results indicated that the upregulation of EHMT2 in T2DM-BMSCs were responsible for the weaker osteogenic potency, which was exerted through the downregulation of LINC00657.

### LINC00657 promoted osteogenic differentiation of T2DM-BMSCs by sponging miR-204-5p

**Figure 3:**
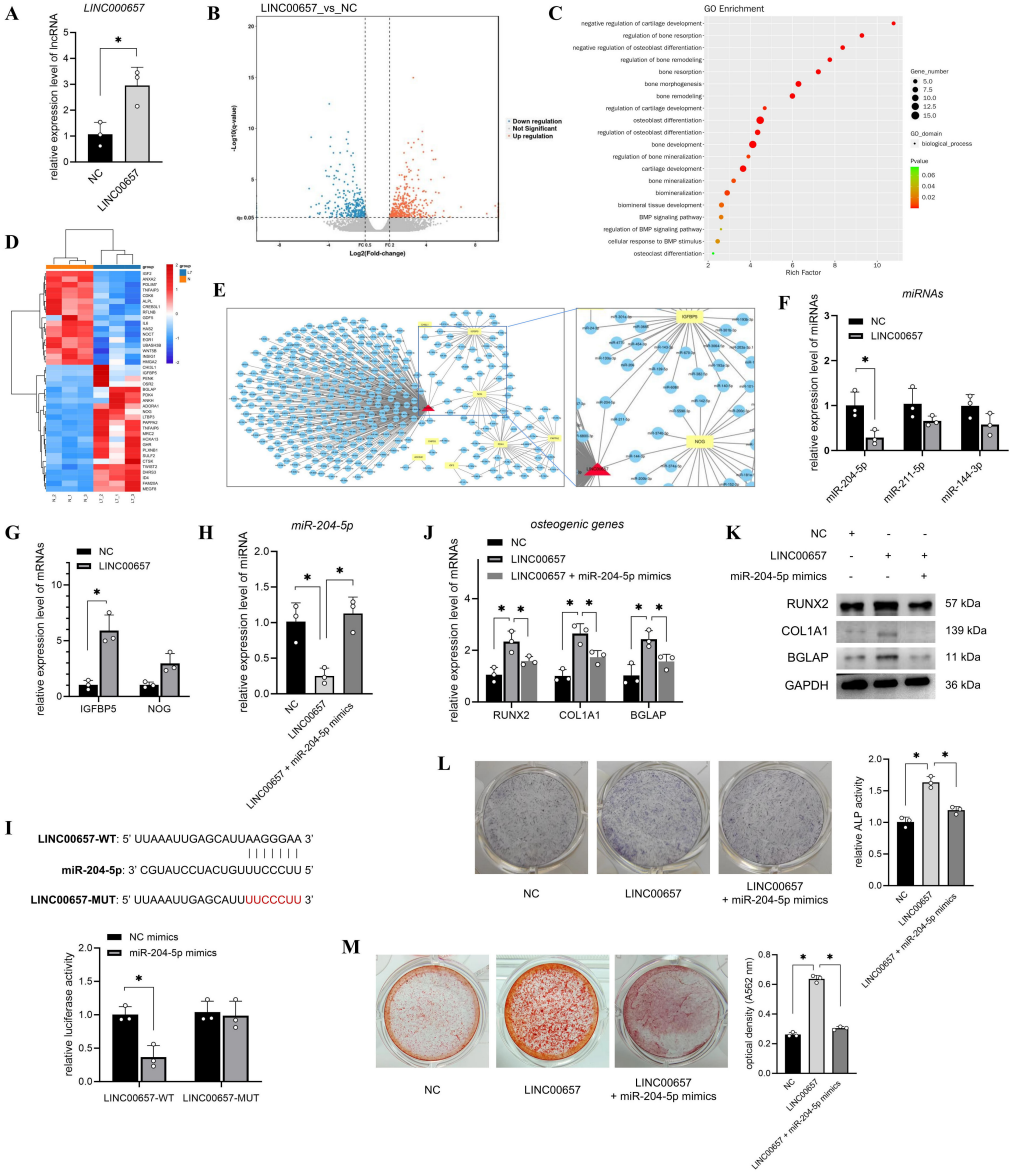
LINC00657 promoted osteogenic differentiation of T2DM-BMSCs by sponging miR-204-5p. **(A)** T2DM-BMSCs were transfected with the LINC00657 overexpression vector, and qPCR was performed to detect LINC00657 expression. **(B)** Volcano plot of DEGs in LINC00657-overexpressed T2DM-BMSCs. **(C)** GO enrichment of all DEGs was analyzed, and 20 osteogenically relevant GO terms were screened and presented in a bubble chart. **(D)** Heatmap of 40 DEGs enriched in osteogenic relevant GO terms screened in T2DM-BMSCs. T: T2DM group, C: CON group. **(E)** ceRNA network of LINC00657 and the top 10 DEGs. **(F, G)** qPCR analysis of the expression levels of miRNAs **(F)** and DEGs **(G)** in T2DM-BMSCs overexpressed LINC00657. **(H)** T2DM-BMSCs were transfected with the LINC00657 overexpression vector or co-transfected with miR-204-5p mimics, and qPCR was performed to detect the expression levels of miR-204-5p. **(I)** Dual-luciferase reporter assay was performed to detect the binding capacity of LINC00657 to miR-204-5p. **(J–M)** T2DM-BMSCs overexpressing LINC00657 (and miR-204-5p) were induced for 7 days, osteogenic genes (*RUNX2, COL1A1*, and *BGLAP*) were detected using qPCR **(J)** and western blotting **(K)**, ALP activity was detected by staining and activity quantification **(L)**, and mineralization level was detected using ARS staining after 14 days of induction **(M)**. ARS, Alizarin Red staining; DEGs, differentially expressed genes; BMSCs, bone marrow-derived mesenchymal stem cells; T2DM, type 2 diabetes mellitus. *, P<0.05.

To screen the miRNAs sponged by LINC00657, the miRNA of *LINC00657* was predicted and 296 miRNAs were obtained. Next, we focused on the mRNAs regulated by LINC00657. T2DM-BMSCs overexpressing LINC00657 were confirmed using qPCR (**Figure 3A**) and 433 DEGs (FC > 2, P < 0.05) were screened using transcriptome sequencing (**Figure 3B**). Next, osteogenically relevant GO terms were screened, including 20 biological processes (**Figure 3C**) and 40 DEGs enriched in these processes (**Figure 3D**, **Supplementary Table S3**). Next, the miRNAs of the top 10 DEGs were predicted and a ceRNA network of LINC00657 was constructed. As shown in **Figure 3E**, three shared miRNAs (*miR-204-5p, miR-211-5p*, and *miR-144-3p*) and two potential target genes (*IGFBP5* and *NOG*) were identified. qPCR validation indicated that *miR-204-5p* expression was significantly reduced (p < 0.05), whereas *IGFBP5* mRNA levels were increased by approximately five folds in the LINC00657-overexpression group (**Figures 3F,G**). Downregulation of *miR-204-5p* and upregulation of *IGFBP5* indicated that they might act downstream of LINC00657.

Next, T2DM-BMSCs were co-transfected with miR-204-5p mimics to investigate their roles in osteogenic differentiation. The qPCR results indicated that co-transfection with miR-204-5p reversed its downregulation compared with the LINC00657 overexpression group (**Figure 3H**). A dual-luciferase reporter assay showed that the miR-204-5p mimic decreased the luciferase activity of cells transfected with the LINC00657 wild-type vector but not the mutant vector (**Figure 3I**), indicating that LINC00657 could sponge miR-204-5p. Additionally, co-transfection with miR-204-5p mimics inhibited the expression of osteogenic markers, including RUNX2, COL1A1, BGLAP, ALP, and calcification, compared with that of the LINC00657 overexpression group (**Figures 3J–M**).

### miR-204-5p suppressed the osteogenic differentiation of T2DM-BMSCs by sponging IGFBP5

**Figure 4:**
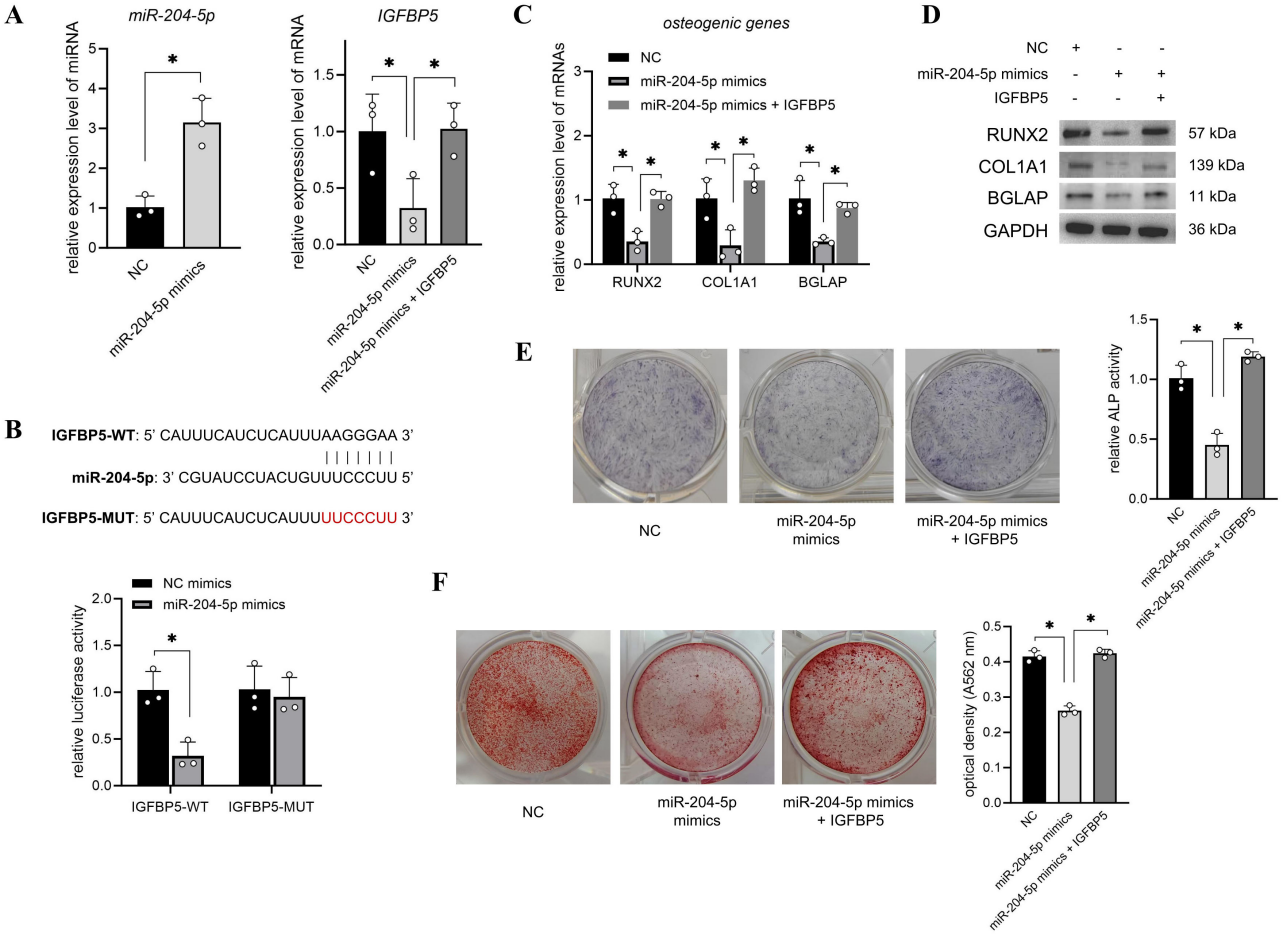
miR-204-5p suppressed the osteogenic differentiation of T2DM-BMSCs by sponging IGFBP5. **(A)** T2DM-BMSCs were transfected with miR-204-5p mimics or co-transfected with an IGFBP5 overexpression vector, and qPCR was performed to detect the expression levels of miR-204-5p and IGFBP5. **(B)** A dual-luciferase reporter assay was performed to detect the binding capacity of miR-204-5p and IGFBP5. **(C–F)** miR-204-5p (and IGFBP5)-overexpressed T2DM-BMSCs were induced for 7 days; osteogenic genes (*RUNX2, COL1A1*, and *BGLAP*) were detected using qPCR **(C)** and western blotting **(D)**; ALP activity was detected using staining and activity quantification **(E)**; and mineralization levels were detected using ARS staining after 14 days of induction **(F)**. BMSCs, bone marrow-derived mesenchymal stem cells; T2DM, type 2 diabetes mellitus. *, P<0.05.

Next, T2DM-BMSCs were transfected with miR-204-5p mimics or co-transfected with an IGFBP5 overexpression vector. The qPCR results indicated that miR-204-5p levels were enhanced and IGFBP5 levels were decreased in the miR-204-5p overexpression group compared with those in the NC group (**Figure 4A**). Further, co-transfection with the IGFBP5 overexpression vector caused a significant increase in IGFBP5 expression (**Figure 4A**, p < 0.05), indicating a potential sponge relationship between miR-204-5p and IGFBP5. The results of the dual luciferase reporter assay showed that luciferase activity was significantly decreased in the IGFBP5-WT group (p < 0.05), whereas it did not change in the mutant group (**Figure 4B**). These results confirm the sponging relationship between the two RNAs. Furthermore, we found that the overexpression of miR-204-5p inhibited the osteogenic potential of T2DM-BMSCs, and co-overexpression of IGFBP5 reversed this inhibitory effect (**Figures 4C–F**).

### UNC0638 alleviated the osteoporosis level of DOP rat through regulating LINC00657/miR-204-5p/IGFBP5 pathway

**Figure 5:**
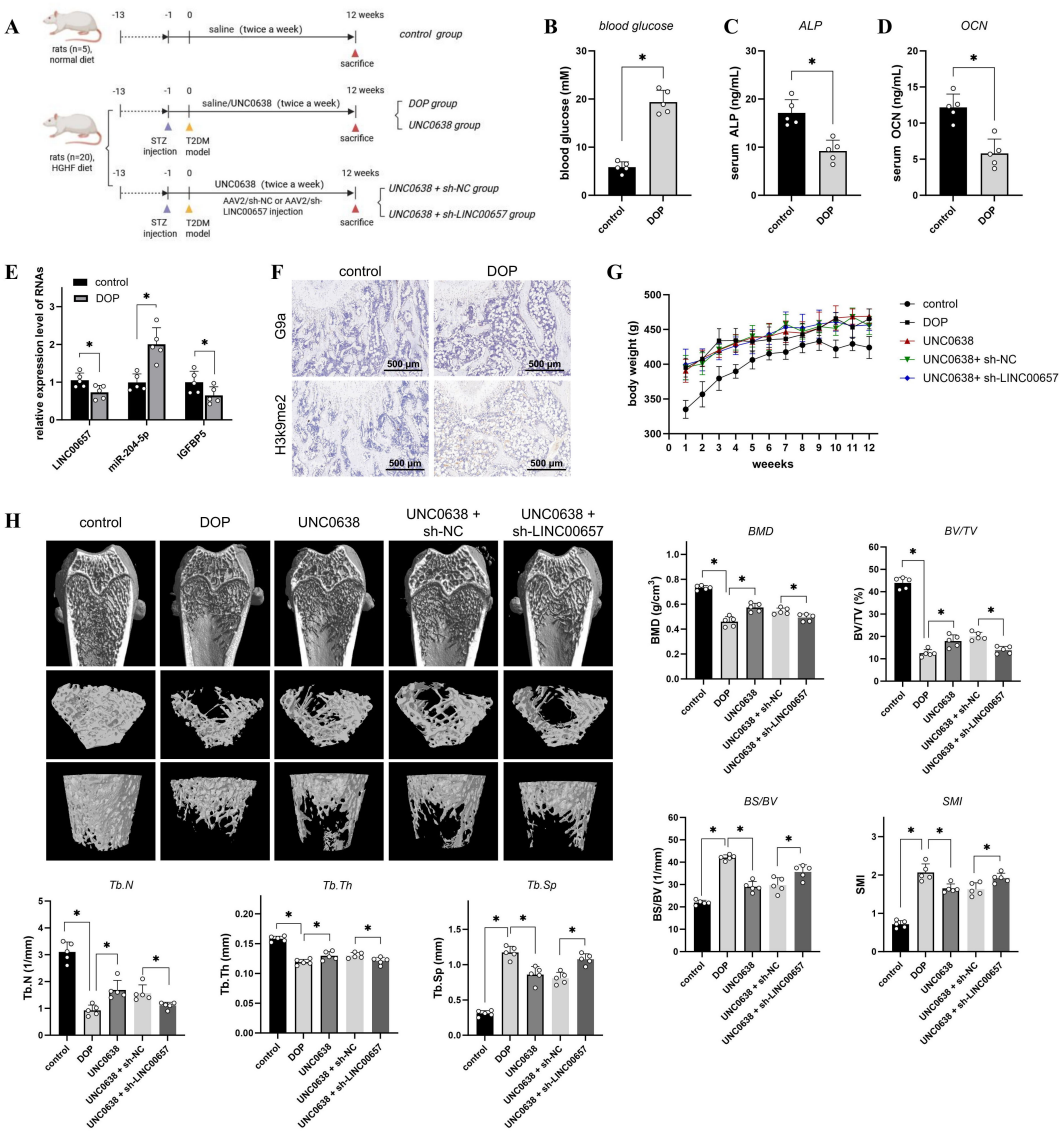
The effect of UNC0638 and knockdown of LINC00657 on the microarchitecture of distal femur in rat DOP model. **(A)** Schematic flow chart of the animal assay. **(B–D)** Rats were fed with high glucose and high fat diet for 12 weeks, followed by STZ injection, and the blood glucose **(B)**, serum ALP **(C)**, and serum OCN **(D)** of rats in two groups were detected. **(E)** qPCR was used to detect expression levels of LINC00657, miR-204-5p, and IGFBP5 in two groups. **(F)** IHC was used for detection of G9a and H3K9me2 in DOP and control rats. Scale: 500 μm. **(G,H)** DOP rats were treated with UNC0638 or knockdown of LINC00657, the body weight were recorded weekly **(G)** and micro-CT analysis **(H)** was performed detect the microarchitecture of distal femur and bone indexes (BMD, BV/TV, Tb.Th, Tb.N, BS/BV, Tb.Sp and SMI). HGHF: high glucose and high fat; BMD, bone mineral density; BS/BV, bone surface/volume ratio; BV/TV, trabecular bone volume; DOP, diabetic osteoporosis; IHC, immunohistochemistry; Tb.Th, trabecular thickness; Tb.N, trabecular number; Tb.Sp, trabecular separation; SMI: structure model index. *, P<0.05.

**Figure 6:**
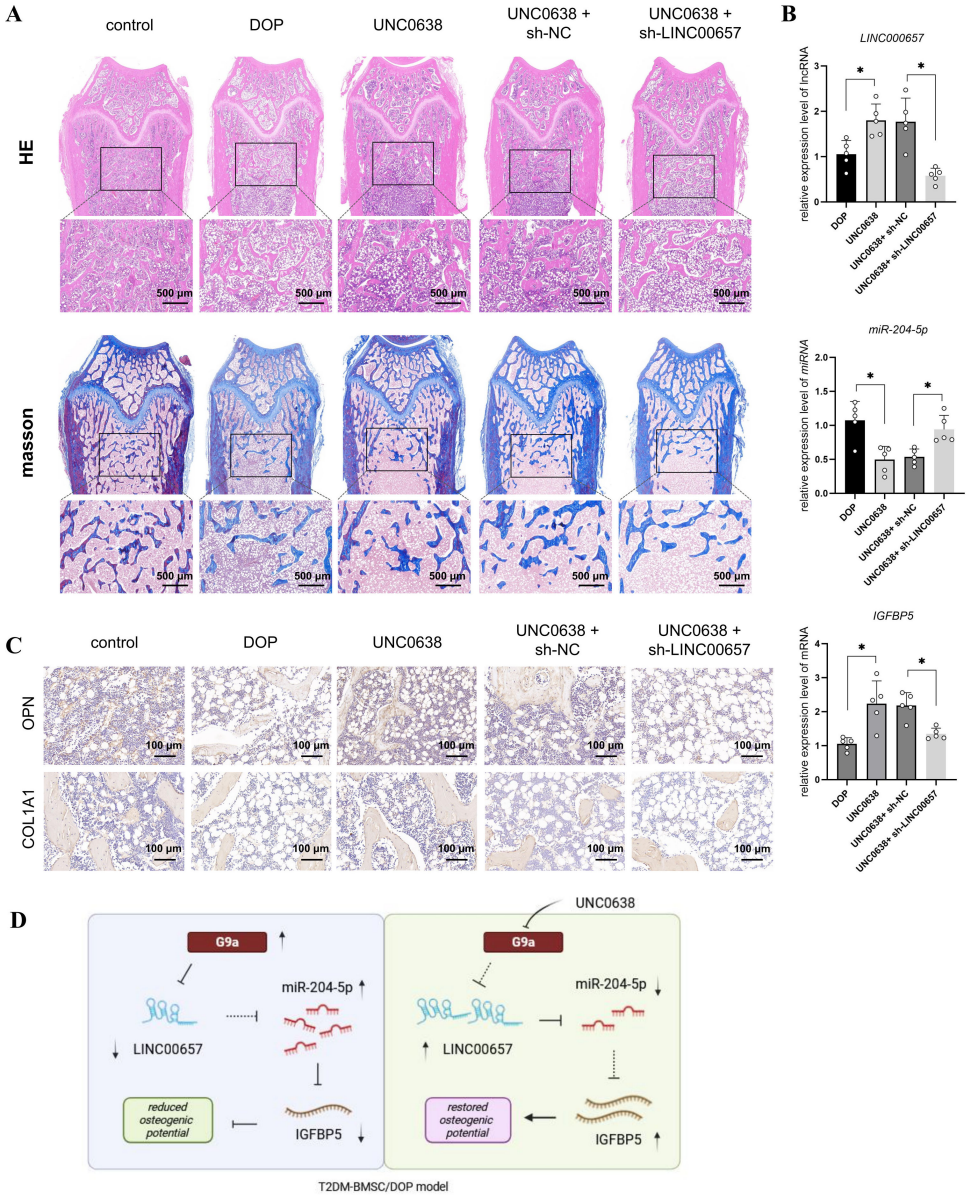
UNC0638 alleviated the osteoporosis level of DOP rat through regulating LINC00657/miR-204-5p/IGFBP5 pathway. **(A)** DOP rats were treated with UNC0638 or knockdown of LINC00657, H&E and Masson’s staining were performed to detect the changes of trabecular bone structure of distal femur (scale: 500 μm); **(B)** qPCR was used to detect expression levels of LINC00657, miR-204-5p, and IGFBP5; **(C)** IHC detection of expression level of osteogenic marker (OPN and COL1A1), scale: 100 μm. DOP, diabetic osteoporosis; H&E, hematoxylin and eosin; IHC, immunohistochemistry. **(D)** Mechanism diagram of UNC0638. *, P<0.05.

The flow chart of animal assay was presented in **Figure 5A**. We established a rat model of DOP to investigate the *in vivo* effects and mechanisms of UNC0638 treatment. As shown in **Figure 5B**, the blood glucose level of rats in the DOP group was >16.7 mM, which was significantly higher than that of rats in the control group (p < 0.05). Serum ALP and OCN levels in the DOP group were reduced by approximately 50% compared with those in the control group (**Figures 5C, D**). Furthermore, the bone trabecular structure became fewer and sparser, based on micro-CT analysis (**Figure 5H**), and H&E and Masson staining (**Figure 6A**). Compared with the control group, BMD, BV/TV, Tb.Th, and Tb.N were significantly decreased, and BS/BV, Tb.Sp and SMI were increased in the DOP group (**Figure 5H**, p < 0.05). In addition, OPN and COL1A1 levels decreased in the distal femur of DOP rats (**Figure 6C**). These changes indicated that the DOP model was successfully established.

Next, G9a and H3K9me2 expression was detected by IHC and enhanced in the distal femur of DOP rats (**Figure 5F**), consistent with the *in vitro* results. The qPCR results indicated a significant decrease of LINC00657 and IGFBP5 expression, and an increase of miR-204-5p level, in the DOP group in comparison with the control group (**Figure 5E**, p < 0.05). After 3 months of treatment with UNC0638, no significant alterations in body weight were observed among the three groups, compared with the DOP group (**Figure 5G**). Osteoporosis progression was significantly inhibited by UNC0638, including significantly increased levels of BMD, BV/TV, Tb.Th, and Tb.N, and decreased levels of BS/BV, Tb.Sp and SMI compared with those in the DOP group (**Figure 5H**, p < 0.05). The bone trabecular structure, and OPN and COL1A1 levels in the distal femur increased after UNC0638 treatment (**Figures 5H**, **6A, C**). Further, qPCR results showed that LINC00657 and IGFBP5 were notably enhanced, and miR-204-5p was reduced in the femoral head of DOP rats after treatment with UNC0638 (**Figure 6B**, p < 0.05). This indicated that the *in vivo* anti-osteoporotic effect of UNC0638 was related to the upregulation of LINC00657 and its downstream molecules, miR-204-5p and IGFBP5.

DOP rats treated with UNC0638 were also intravenously injected with lentiviral sh-LINC00657 or sh-NC. The qPCR results confirmed that LINC00657 levels were lower in the UNC0638 + sh-LINC00657 group compared with that in the UNC0638+ sh-NC group (**Figure 6B**). Micro-CT, and H&E, and Masson staining results showed that knockdown of *LINC00657* significantly inhibited the anti-osteoporosis effect of UNC0638, including a decrease in four bone indices (BMD, BV/TV, Tb.Th, and Tb.N) and an increase in three indices (BS/BV, Tb.Sp and SMI) (**Figure 5H**, p < 0.05; and **Figure 6A**). The IHC results indicated that OPN and COL1A1 were both decreased after LINC00657 knockdown (**Figure 6C**). Furthermore, downregulation of LINC00657 caused a significant increase in miR-204-5p and a decrease in IGFBP5 in the femoral head (**Figure 6B**, p < 0.05). The above results indicate that the upregulation of LINC00657 was responsible for the anti-osteoporosis effect of UNC0638 in DOP rats, and UNC0638 treatment also regulated the expression of miR-204-5p and IGFBP5.

In summary, G9a inhibited the osteogenic potential of T2DM-BMSCs by regulating the LINC00657/miR-204-5p/IGFBP5 axis and UNC0638 reversed DOP by inhibiting G9a/LINC00657/miR-204-5p/IGFBP5 axis (**Figure 6D**).

## 4 Discussion

The considerable number of individuals with DM and high prevalence of osteoporosis in these populations necessitate greater attention to DOP. The relationship between DM and osteoporosis is complex and DOP is a secondary form of osteoporosis. Generally, multiple factors, including chronic hyperglycemia, insulin resistance, and AGEs, lead to the activation of oxidative stress and inflammation, thereby causing dysregulation of bone metabolism and systemic bone loss (44, 45). For the BMMSCs, hyperglycemia activates p53 pathway, thereby resulting in senescence, and weakened proliferation and osteogenic differentiation abilities (27). Accelerated ROS inhibits osteogenic differentiation and promotes adipogenesis through regulating AKT-mTOR pathway (28). Hyperglycemia also upregulates PPARγ expression of BMMSC, thus inhibiting RUNX2 expression and promoting adipogenesis (46). Activation of TLR4/NF-κB pathway inhibits Wnt/β-catenin pathway and further blocks osteogenic differentiation (47). Currently, understanding the mechanism underlying the reduced osteogenic potential of BMSCs in T2DM from the perspective of epigenetic regulation (48, 49) is of great importance for the development of potential novel therapeutic strategies for DOP.

The present study found that G9a was increased in T2DM-BMSCs and UNC0638 improved the osteogenic potential of T2DM-BMSCs. The positive effect of the G9a inhibitor was not contradictory with previous studies (20–22). Firstly, the deletion of G9a causes incomplete ossification (20), indicating that G9a is necessary for this process. Secondly, the administration dose of G9a inhibitors may cause different effects. The IC50 values of A366, BIX01294, and UNC0638 against G9a are 3.3 nM, 1.9 μM, and <15 nM, respectively. A366 showed a higher affinity than UNC0638. The *in vivo* dosage of A366 is 25 mg/kg (21), which is five times that of the dose used in our rat DOP model. We also found that, higher dose (> 1 μM) of UNC0638 is cytotoxic. G9a protein level was enhanced in T2DM-BMSCs and the DOP model. Moderate inhibition of G9a with a lower dose of UNC0638 (0.1, 0.5 μM) benefits to the recovery of osteogenic potential. Additionally, 1.5 μM of BIX01294 suppresses osteoclast differentiation of Raw264.7 cells (22), indicating that inhibition of G9a benefits to the osteogenesis, consistent with our findings.

Recent studies have also identified the role of lncRNAs in the progression of DOP (31, 32), and the crosstalk between lncRNAs and G9a (33, 35). Our previous study found that lncRNAs are significantly changed in T2DM-BMSCs compared with that in CON-BMSCs (24); therefore, we inferred that the dysregulation of lncRNAs might be related to the increase in G9a. LINC00657 was screened after qPCR validation and the present study confirmed its pro-osteogenic role in T2DM-BMSCs. Currently, *LINC00657* is considered an oncogene in tumorigenesis, including cervical cancer (50–52) and another study reported its opposite role in cervical cancer progression (53). To date, the role of LINC00657 in osteoporosis is less understood and only one report demonstrated its proosteogenic role in BMMSC via the miR-144-3p/BMPR1B axis (54), which is consistent with our results to some extent. In addition, serum LINC00657 levels were significantly enhanced in patients with diabetes without a stroke compared with that in healthy controls but were decreased in patients with diabetes with a stroke (55). This report indicates a negative correlation between LINC00657 in patients with diabetes with complications, similar to the downregulation of LINC00657 in T2DM-BMSCs to some extent. The relationship between serum LINC00657, DOP, and osteoporosis requires further investigation. The present study revealed the regulatory effect of G9a on LINC00657; however, the potential mechanism was not investigated, which is one of the limitations of this study.

MiRNAs are also responsible for the reduced osteogenic potential of BMSC in diabetic environment. MiR-491-5p is decreased in jawbone marrow MSC of patients with T2DM, and overexpressing miR-491-5p alleviates osteogenic potential by regulating SMAD/RUNX2 pathway (56). High glucose induces the increased level of miR-493-5p, thus preventing osteogenic differentiation by targeting ZEB2 (29). Therefore, we focused on the miRNAs of LINC00657 and their potential target genes. Generally, bioinformatics analysis indicates hundreds of matched lncRNA and miRNAs, which makes their selection and validation difficult. The combination of different datasets or tools to screen for overlapping miRNAs is a common method to screen candidate miRNAs (54). However, the precise roles of the final miRNA target genes in the relevant phenotypes remain unclear. Therefore, a different approach was adopted in this study. LINC00657 was overexpressed in T2DM-BMSCs and DEGs regulated by LINC00657 were identified. GO enrichment was performed to screen for genes involved in osteogenesis. Finally, by combining the ceRNA network construction, the number of candidate miRNAs was reduced from hundreds to several. This approach is beneficial for filtering the least relevant miRNAs and identifying the most effective miRNAs and target genes. The present study showed that LINC00657 promotes the osteogenic differentiation of T2DM-BMSCs by sponging miR-204-5p, indicating an inhibitory role of miR-204-5p. Similarly, other studies have revealed an inhibitory effect of miR-204-5p on the osteogenic differentiation of ankylosing spondylitis fibroblasts and valve interstitial cells (57, 58).

IGFBP5 belongs to the insulin-like growth factor-binding protein family (IGFBPs). They were initially thought to be regulatory proteins of IGFs in the blood circulation, primarily as a means of bidirectional regulation of IGF function during environmental changes (59). However, recent studies have revealed that IGFBP5 possesses a multitude of functions independent of IGFs (60), including the suppression of lipid deposition (61), aging (62), promotion of tumor progression (63), and osteogenesis (64). IGFBP5 has been identified as an important driver in the early osteogenic differentiation of BMMSC (64), and the upregulation of IGFBP5 is beneficial for the osteogenesis and dentinogenesis of dental pulp stem cells (65, 66). Consistently, the present study found that the overexpression of IGFBP5 significantly reversed the inhibitory effect of miR-204-5p.

STZ injection is considered one of the most common methods for establishing a DOP animal model (67). The present study successfully constructed a rat DOP model through STZ injection and an HGHF diet, and intraperitoneal injection of UNC0638 effectively inhibited the progression of DOP, consistent with the *in vitro* results. Intraperitoneal injection of a G9a inhibitor is a widely used method (21, 39, 40). Some studies have indicated that a single injection of a G9a inhibitor is effective against renal fibrosis and acute myocardial infarction-induced damage (39, 40), indicating the powerful and constant effect of a G9a inhibitor. In the present study, the administration frequency of the G9a inhibitor was increased to twice a week to prevent the progression of osteoporosis induced by hyperglycemia and the HGHF diet. Further knockdown of LINC00657 in the DOP model suppressed the effectiveness of UNC0638 treatment, confirming that LINC00657 is indeed an effective lncRNA.

There are some limitations in the present study. Firstly, only five rats were used per group in the animal assay, and a larger sample size (n=6-8) would enhance the reliability and persuasiveness of our findings. Secondly, the effective dose range of UNC0638 for DOP requires confirmation in future studies to ensure its effectiveness. The downstream pathways of LINC00657/miR-204-5p/IGFBP5 axis were not investigated and also require further study.

In summary, the *in vitro* and *in vivo* experiments demonstrated that G9a inhibited the osteogenic potential of T2DM-BMSCs by regulating the LINC00657/miR-204-5p/IGFBP5 pathway. Our study provides a novel mechanism for the reduced osteogenic potential of T2DM-BMSCs and UNC0638 can be developed as a potential agent for DOP treatment.

## Data Availability

The original contributions presented in the study are included in the article/**Supplementary Material**. Further inquiries can be directed to the corresponding author.
